# Cross-cultural adaptation and reliability assessment of the Ecuadorian youth risk behavior survey in Ecuadorian adolescents

**DOI:** 10.3389/fped.2026.1937879

**Published:** 2026-09-21

**Authors:** Rosero Alejandra, Soria Rocío, González Paloma, Abarca Ximena, Velasco María, Fors Martha

**Affiliations:** 1 Ministerio de Educación del Ecuador, Quito, Ecuador; 2 Bibliotecas - Universidad de Las Américas, Quito, Ecuador; 3 Universidad de Las Américas, Quito, Ecuador; 4 Universidad de Las Américas, Facultad de Ciencias de la Salud, Escuela de Medicina, Grupo Onehealth, Quito, Ecuador

**Keywords:** adolescent health, child/adolescent health, college health, community health, crosscultural health, environmental health, health behavior, health promotion

## Abstract

**Background:**

Reliable and culturally adapted instruments are essential for monitoring health-risk behaviors among adolescents and supporting evidence-based public health interventions. The Youth Risk Behavior Survey (YRBS) is widely used internationally; however, no culturally adapted version had previously been evaluated for use among Ecuadorian adolescents.

**Objective:**

To assess the internal consistency and test-retest reliability of the Ecuadorian adaptation of the Youth Risk Behavior Survey (EYRBS). A cross-sectional study with a longitudinal test-retest component was conducted among 356 adolescents aged 12–17 years from public schools in Ecuador. The Spanish version EYRBS was administered online. To evaluate temporal stability, 67 participants completed the questionnaire a second time 15 days after the initial administration. Internal consistency was assessed using Cronbach's alpha coefficient and McDonald omega. Test-retest reliability was evaluated using the intraclass correlation coefficient (ICC) based on a two-way mixed-effects model with absolute agreement.

**Results:**

The final version of the EYRBS consisted of 51 items organized into two domains: Social Aspects and Health Aspects. The overall Cronbach's alpha coefficient was 0.686, while the Social Aspects and Health Aspects domains achieved alpha values of 0.616 and 0.644, respectively. Internal consistency, assessed via McDonald's omega (*ω*), yielded acceptable values, with 0.680 for Social Aspects, 0.661 for Health Aspects, and 0.780 for the overall scale. Test-retest reliability was excellent, with an overall ICC of 0.79 (95% CI: 0.66–0.89), and domain-specific ICCs of 0.85 for Social Aspects and 0.78 for Health Aspects.

**Conclusions:**

The EYRBS demonstrated acceptable internal consistency and excellent temporal stability, providing initial evidence of reliability. Structural validity and advanced psychometric evaluations are intentionally deferred to a subsequent, separate investigation.

## Highlights

- The Ecuadorian adaptation of the Youth Risk Behavior Survey (EYRBS) demonstrated excellent test-retest reliability and acceptable-to-questionable internal consistency among Ecuadorian adolescents, supporting its initial use for epidemiological surveillance.
- The adapted EYRBS enables a culturally and linguistically appropriate assessment of health and social risk behaviors tailored to the context of adolescent populations in Ecuador.
- Practitioners and policymakers can leverage EYRBS data to design targeted, evidence-based public health interventions aimed at improving adolescent health and well-being.

## Introduction

The significance of establishing a mechanism to comprehend and ascertain the genuine state of various health risk-related matters is associated with the ability to monitor temporal trends, recognize emerging issues, and plan and assess programs aimed at addressing risky behaviors (1). The Youth Risk Behavior Survey (YRBS) serves this purpose and was created by the Centers for Disease Control and Prevention (CDC) in the United States for implementation within educational institutions, serving as an international benchmark for evaluating adolescent risky behaviors (1).

YRBSS monitors a wide range of health-related behaviors, including risky behaviors, among young people and young adults. The data collected through the YRBS provide valuable insights into the prevalence of these behaviors, trends over time, and potential risk factors.

In Ecuador, there is no systematic heath risk behavior survey for the adolescent population. The cross-cultural adaptation of the questionnaire for the Ecuadorian context is important because they provide valuable information for a comprehensive understanding of the prevailing conditions. Having access to accurate information is essential for the development of evidence-based public policies aimed at addressing these issues.

The study focused on assessing the temporal stability and reliability of the instrument among Ecuadorian adolescents. The data used in this study were obtained through the official administration of the EYRBS, which was translated and culturally adapted following a systematic methodology (2, 3).

The cultural adaptation of the Ecuadorian Youth Risk Behavior Surveillance Survey (EYRBS) will provide practitioners with a robust tool to assess risky behaviors in adolescents while considering cultural and contextual nuances. This questionnaire ensures that questions reflect the realities and cultural dynamics of Ecuadorian adolescents, improving the accuracy of responses and relevance of data for local interventions and offers practitioners confidence in measuring health-related behaviors, enabling consistent identification of at-risk youth.

Although the Youth Risk Behavior Survey (YRBS) has been widely used internationally, its direct application in Ecuador may not adequately capture the country's linguistic, cultural, and social diversity. Ecuador is characterized by a multicultural population, including Indigenous, Afro-Ecuadorian, Montubio, Mestizo, and other ethnic groups distributed across the Coast, Highlands, Amazon, and Insular regions. These cultural differences may influence adolescents' interpretation and reporting of health-risk behaviors, highlighting the need for a culturally adapted version that ensures semantic, conceptual, and contextual equivalence before reliability assessment.

The aim of this study was to evaluate the reliability of the Ecuadorian Youth Risk Behavior Survey (EYRBS), following its cross-cultural adaptation, by assessing its internal consistency and temporal stability among Ecuadorian adolescents. Given the multidimensional and epidemiological surveillance nature of the instrument, the scope of this paper is strictly limited to cross-cultural adaptation and reliability assessment (internal consistency and temporal-stability). Comprehensive structural validity analyses (such as EFA and CFA) require distinct methodological considerations and sample planning, and are deferred to a subsequent validation study.

## Methods

### Study design and participants

This methodological study evaluated the reliability of the Ecuadorian version of the Youth Risk Behavior Survey (EYRBS) following its cross-cultural adaptation. A cross-sectional design was used to assess internal consistency, complemented by a longitudinal test–retest component to evaluate temporal stability. Participants were recruited from 12 public educational institutions located in six provinces across the Coast, Highlands, and Amazon regions of Ecuador, including both urban and rural settings. Schools were selected based on geographic representation, availability of internet connectivity, and the presence of Student Counseling Department (DECE) professionals to facilitate data collection. Within each participating school, students from the eighth grade of basic education through the third year of high school were selected using simple random sampling with proportional representation by sex. For the test–retest reliability assessment, six students (three females and three males) were initially selected from each participating school (*n* = 72). Of these, 67 completed the second administration of the questionnaire 15 days after the baseline assessment and were included in the temporal stability analysis. The inclusion criteria were age between 12 and 17 years old. Participation in the study was entirely voluntary. Students provided assent, and their legal guardians gave informed consent for their involvement. Participants were from institutional public units located in 6 provinces of the country. The survey was administered online in a self-administered format. A total of 425 adolescents from 12 public educational institutions located in the Coast, Highlands, and Amazon regions of Ecuador were initially recruited. After excluding 69 questionnaires with incomplete responses, the final baseline sample comprised 356 participants, who were included in the internal consistency analyses. For the test–retest reliability assessment, 72 students (six from each participating school) were selected. The questionnaire was re-administered 15 days later, and five participants were excluded because of incomplete paired responses, resulting in a final test–retest sample of 67 adolescents for the intraclass correlation coefficient (ICC) analyses. Therefore, analyses of internal consistency were performed using data from the initial administration, whereas test-retest reliability analyses were conducted using paired responses from the 67 participants who completed both assessments (Figure 1).

**Figure 1:**
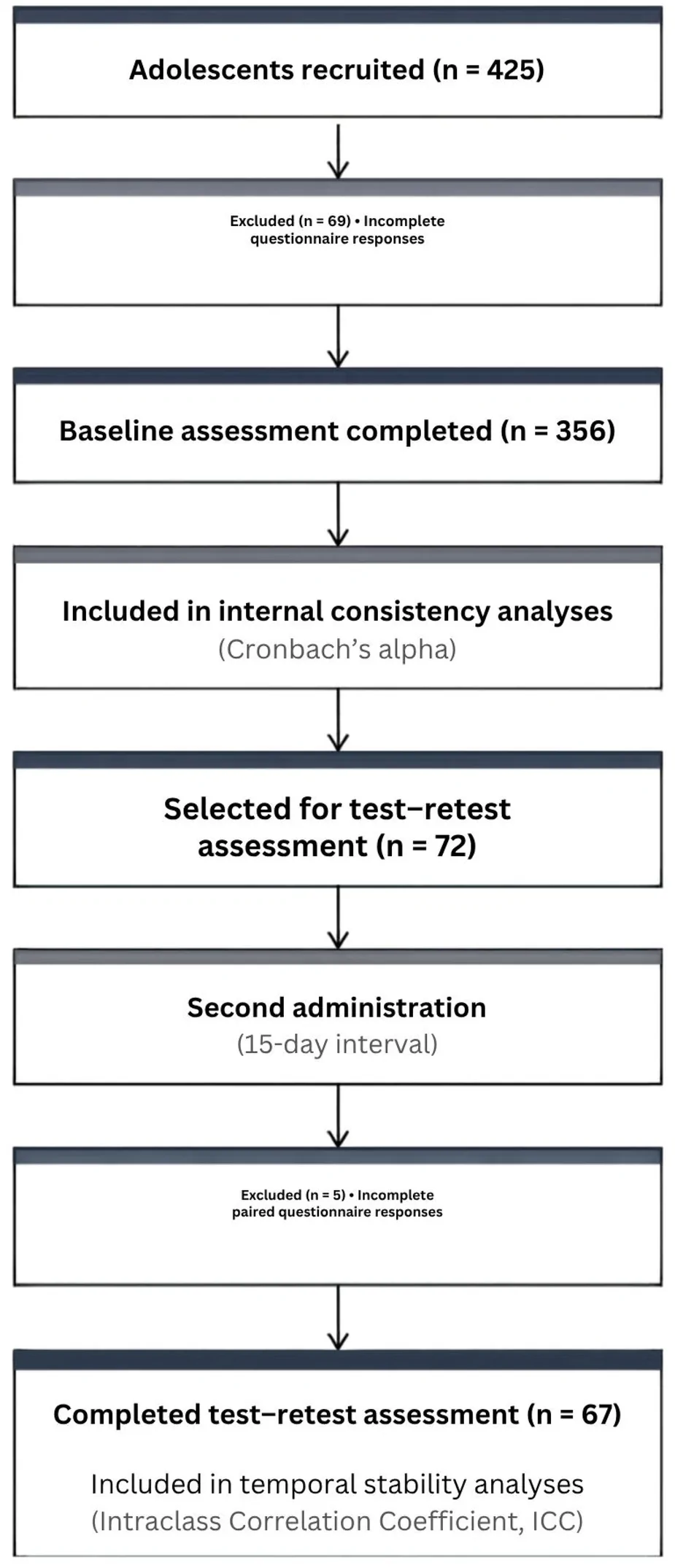
Participant flow from initial recruitment through the baseline assessment and test–retest reliability phases of the Ecuadorian youth risk behavior survey (EYRBS).

### Measurement instrument

The original Youth Risk Behavior Survey (YRBS), first developed by the Centers for Disease Control and Prevention (CDC) in 1991, consisted of 75 questions. Four questions collected demographic and general information, while the remaining items assessed multiple risk behavior domains, including unintentional injuries and violence (17 items), tobacco use (9 items), alcohol and other drug use (14 items), sexual behaviors (8 items), dietary behaviors (7 items), physical inactivity (8 items), weight management (4 items), HIV-related behaviors (3 items), and other health-related topics (1 item) (4, 5).

The Ecuadorian Youth Risk Behavior Survey (EYRBS), developed in 2022 through a process of cultural adaptation and contextualization. To ensure content validity and cultural relevance, the adapted questionnaire underwent an expert review process. The panel included seven professionals from the fields of medicine, nursing, psychology, public health, and education, all with at least five years of experience in research, clinical practice, or academic work related to adolescents.

The initial Ecuadorian version of the questionnaire comprised 113 questions, including core YRBS items, demographic variables, administrative fields, anthropometric measurements, contextual questions, and skip-pattern items required for questionnaire administration. The reduction to 51 items did not correspond to a statistical item reduction process but to the identification of the core behavioral items included in the reliability assessment. Administrative variables, participant identifiers, demographic and anthropometric questions, and routing items were excluded because they do not measure health-risk behaviors and were therefore outside the scope of the analyses. Consequently, the final analytical version consisted of 51 items grouped into the Social Aspects and Health Aspects domains. The final Spanish version was administered online between August and October 2022. All questions, except those related to height, weight, and ethnicity, were multiple-choice items with a single response option, following the format of the original YRBS. For the purposes of the psychometric analyses, the 51 core behavioral items were organized into two broad conceptual domains according to the thematic structure of the original YRBSS and their relevance to adolescent health surveillance in Ecuador.1. Social Aspects: This domain comprised behavioral factors primarily related to the adolescents' social environment and interpersonal interactions. It included items derived from the original YRBSS domains addressing behaviors contributing to unintentional injuries and violence, such as the use of seat belts and helmets, physical fighting, bullying, and experiences of violence, as well as tobacco use, alcohol consumption, and the use of illicit drugs and other psychoactive substances. These behaviors were grouped because they represent major social and behavioral risk factors associated with injury, violence, and substance use during adolescence. 2. Health Aspects: This domain comprised behaviors directly related to health promotion and disease prevention. It included items from the original YRBSS domains assessing sexual behaviors associated with unintended pregnancy and sexually transmitted infections (including HIV), dietary behaviors, and physical activity and sedentary behaviors. These components evaluate lifestyle habits that are strongly associated with the prevention of chronic diseases and the promotion of adolescent health.

Additional questions addressing child labor and home abandonment were incorporated because these issues represent important social challenges affecting Ecuadorian adolescents and are highly relevant to the national context.

The final analytic version therefore consisted of 51 items organized into two broad domains: Health Aspects (22 questions) and Social Aspects (29 questions).

To facilitate administration and interpretation within the Ecuadorian context, the original YRBS domains were regrouped into these two broader dimensions. The Health Aspects included items related to sexual health, nutrition, and physical activity, whereas the Social Aspects included items related to violence, tobacco use, alcohol consumption, and other drug-related behaviors.

### Cross cultural adaptation

The Ecuadorian version of the Youth Risk Behavior Survey (EYRBS) was developed following internationally accepted guidelines for the cross-cultural adaptation of health questionnaires. The process included forward translation from English into Spanish by two independent certified bilingual translators, synthesis of both versions, back-translation into English by two independent native English-speaking translators, and reconciliation of discrepancies by the research team. Subsequently, a multidisciplinary expert committee comprising specialists in adolescent health, psychology, nursing, medicine, public health, and linguistics evaluated semantic, idiomatic, conceptual, and cultural equivalence, leading to the adaptation of selected items to the Ecuadorian context. The pre-final version was then pilot-tested in five public educational institutions located in the Coast, Highlands, and Amazon regions to evaluate comprehensibility, acceptability, completion time, and the emotional impact of the questions. Items identified as difficult to understand by at least 15% of participants were reviewed and revised before producing the final Ecuadorian version of the questionnaire used in the present reliability study. The present manuscript focuses on the reliability assessment of the final Ecuadorian version of the questionnaire. Therefore, the cross-cultural adaptation process is summarized here to provide the methodological context, whereas the complete adaptation methodology and its detailed results are reported separately.

### Demographic variables

We also collected information regarding the participants' demographic characteristics, such as age and sex.

### Sample size

The sample size was considered adequate for an initial methodological study focused on evaluating the reliability of the Ecuadorian version of the questionnaire. The primary objective was to assess internal consistency and temporal stability rather than to perform a comprehensive psychometric validation. According to the COSMIN methodological framework, the adequacy of the sample should be interpreted in relation to the measurement properties being evaluated. Therefore, the baseline sample of 356 adolescents and the test–retest subsample of 67 participants were considered appropriate for estimating internal consistency and temporal stability in this initial reliability study (6, 7).

### Statistical analysis

Descriptive statistics were performed for demographic variables. Descriptions of variables were made using tables of frequencies, means, and standard deviations (SDs). The distribution of continuous variables was assessed using the Shapiro–Wilk test. Categorical variables, including sex and ethnicity, were summarized using absolute and relative frequencies, as normality testing is not applicable to nominal categorical variables.

Internal consistency reliability was evaluated using McDonald's omega coefficient () along with its 95% bootstrap confidence intervals, following methodological recommendations for psychometric evaluation. Thresholds for interpretation were categorized as acceptable, good, and excellent (8).

To estimate the internal consistency of the scores, the Cronbach's alpha coefficient was calculated. The following rules were applied: alpha > 0.9 – Excellent, alpha >0.8 – Good, alpha > 0.7 – Acceptable, alpha >0.6 – Questionable, alpha > 0.5 – Poor, and alpha < 0.5 Unacceptable (9).

To measure the test-retest reliability, we included 67 students, and the intraclass correlation coefficient (ICC) and 95% confidence interval were assessed based on the two-way mixed-effects model, absolute agreement, single measures, with 0.70 as the recommended minimum standard. The following criteria were used as quantitative values for the ICCs: below 0.4 (weak reliability), 04-0.6 (moderate reliability), 0.6-0.8 (good reliability) and 0.8-1 (excellent reliability). The present statistical analyses were intentionally limited to the assessment of reliability through internal consistency and temporal stability, which constituted the primary objective of this methodological study following the cross-cultural adaptation of the questionnaire. Consequently, the analyses were not intended to provide a comprehensive psychometric validation of the instrument. Structural validity through exploratory or confirmatory factor analysis was not assessed in the present study. The two-domain organization used for the reliability analysis was established *a priori* on conceptual and content-based grounds, reflecting the thematic structure of the instrument rather than an empirically derived factor solution. Given the multidimensional surveillance purpose of the YRBSS and its conditional skip-pattern items, a global factor analysis of the complete instrument was not considered appropriate. Additional psychometric properties, including structural validity through exploratory and confirmatory factor analyses, construct validity, McDonald's Omega, and Composite Reliability, require larger samples and will be evaluated in a subsequent nationwide psychometric study. All statistical analyses were performed using SPSS Statistics version 26 (IBM Corp., Armonk, NY, USA). All the statistical analyses were performed using SPSS 26 for Windows (IBM Corp., Armonk, NY, USA).

## Results

### Definitive questionnaire

The study included 356 adolescents. The participants were distributed across the country's regions, with the Sierra region comprising 114 participants (32.02%), the Amazonía region comprising 108 participants (30.34%), and the Costa region comprising 91 participants (25.56%), while 43 participants (12.08%) did not report regional information.

The mean age of participants was 14.53 years, with a standard deviation of 1.71 years. A total of 45.0% of the participating students considered themselves to be female and 49.7% male, none of the participants identified themselves as intersex, and 4.5% of the participants did not register any information about their sex.

The years of schooling of the students participating in the study ranged from the eighth grade of general basic education to the twelve grades of high school. Finally, 76.7% of the students self-identified as mestizo, followed by 6.7% who self-identified as white. Similarly, 10.1% of the students did not report this information.

The overall Cronbach's alpha coefficient was 0.686, indicating an acceptable overall internal consistency for an instrument designed to assess multiple dimensions of adolescent risk behavior. Regarding the specific domains, the Social Aspects domain achieved a Cronbach's alpha of 0.616 (classified as questionable to acceptable), and the Health Aspects domain achieved an alpha of 0.644 (acceptable level of internal consistency). The Social Aspects domain yielded an omega of 0.680, while the Health Aspects domain recorded an omega of 0.661. Finally, the complete Total scale obtained a McDonald's omega of 0.780 (Table 1).

**Table 1:** Internal consistency of the EYRBS domains.

Domain	Number of items	Cronbach's Alpha (*α*)	Interpretation	McDonald's *ω*	Interpretation
Social Aspects	22	0.616 (0.516–0.716)	Acceptable/Questionable	0.680 (0.492–0.788)	Acceptable/Questionable
Health Aspects	29	0.646 (0.579–0.707)	Acceptable	0.661 (0.269–0.768)	Questionable
Overall Scale	51	0.686 (0.616–0.749)	Acceptable	0.780 (0.469–0.824)	Acceptable

### Test and retest study

A total of 67 students completed both the first and second questionnaires 15 days apart.

The age of the participating students was also between 12 and 17 years. Approximately 44% of the participating students were female, and 49.3% were male. Four students did not provide sex information. The years of schooling of the students participating in the study ranged from the eighth grade of general basic education to the third grade of high school. A total of 71.6% of the students self-identified as mestizo, followed by 9% who self-identified as white. Similarly, 8 of the students did not report this information.

The overall ICC was 0.79 (95% CI: 0.66–0.89), while the Social and Health domains showed ICC values of 0.85 (95% CI: 0.77–0.91) and 0.78 (95% CI: 0.65–0.87), respectively (Table 2).

**Table 2:** Intraclass correlation coefficient in the dimension of repeatability of questionnaire domains.

Indicator	Total (IC95%)	Social Domain	Health Domain
Intraclass correlation coefficient	0.79 (0.66–0.89)	0.85 (0.77–0.91)	0.78 (0.65–0.87)
*p* value	0.001	0.001	0.001

## Discussion

This study evaluated the reliability of the Ecuadorian adaptation of the YRBSS and demonstrated acceptable internal consistency together with excellent temporal stability among Ecuadorian adolescents. These findings support the use of the EYRBS as a reliable tool for assessing health-related risk behaviors in this population. To the best of our knowledge, this is the first study to validate this scale in Ecuador. The present study was designed to evaluate the reliability of the EYRBS following its cross-cultural adaptation rather than to perform a comprehensive psychometric adaptation. Although reliability constitutes an essential step in instrument development, additional evidence regarding structural validity, construct validity, McDonald's Omega, Composite Reliability, and factorial structure is required before considering the psychometric evaluation complete. These analyses are currently planned as part of a subsequent nationwide adaptation study. In 2007, in Brazil, the study was conducted in person, with the aim of testing the Portuguese version of the questionnaire to investigate the difficulties and suggestions of adolescents in terms of understanding the questions. For its application, the researchers visited the selected educational institutions on two occasions and applied the instruments to 140 adolescents between 14 and 18 years of age (81 females and 59 males) (3).

The overall Cronbach's alpha coefficient of the EYRBS (α=0.686) indicates acceptable internal consistency, while the domain-specific coefficients α=0.616 for Social Aspects and α=0.644 for Health Aspects demonstrate acceptable to questionable internal consistency levels typical for an epidemiological surveillance instrument composed of conceptually heterogeneous behavioral domains. Unlike unidimensional psychological scales designed to measure a single latent construct, the EYRBS intentionally includes diverse health-risk behaviors, which are not expected to exhibit high inter-item correlations. Therefore, these domain-specific results should be interpreted in light of the multidimensional nature and broad surveillance purpose of the instrument rather than against conventional thresholds developed for homogeneous psychometric scales (9, 10).

These findings are consistent with previous adaptations of the YRBS and similar public health surveillance instruments, in which moderate internal consistency coefficients have been considered acceptable given the breadth of behavioral domains evaluated.

Previous adaptations of the YRBSS and related surveillance instruments have reported similar findings. In Brazil, the evaluation of the Portuguese adaptation of the YRBSS included 140 adolescents aged 14–18 years and focused on comprehension difficulties and cultural adaptation of the questionnaire, demonstrating the importance of evaluating adolescents' interpretation of items before large-scale implementation (3). Likewise, a study conducted among Brazilian university students reported adequate internal consistency (Cronbach's *α* = 0.770) and intraclass correlation coefficients ≥0.75, supporting the temporal stability and reliability of the instrument (11).

The reliability indicators observed for the Spanish-adapted version of the questionnaire were acceptable. By identifying specific behaviors prevalent among Ecuadorian adolescents, practitioners can design interventions tailored to address issues such as substance use, school absenteeism, or other risk factors. The instrument's adaptability to various educational contexts supports its use across diverse regions, aiding practitioners in identifying trends and implementing localized prevention strategies.

Although the grouping of the questions in the EYRBS changes in its major themes, the subthemes and the purpose of evaluating each subtheme through its questions and answers are like those of the original YRBSS since only certain issues of form and context change, maintaining the structure and composition of each item and evaluating what it should evaluate. Importantly, the two-domain organization used in the present study should not be interpreted as an empirically derived factorial structure. It was established *a priori* on conceptual and content grounds to facilitate the reliability analysis of the adapted surveillance instrument. Therefore, factor loadings and factorial fit indices were not estimated in this study.

Child labor and home abandonment were incorporated into the Ecuadorian adaptation because these issues represent relevant social challenges within the national context. Their inclusion sought to improve the cultural relevance of the instrument while maintaining consistency with the original purpose of assessing adolescent risk behaviors.

Additionally, an assessment of the internal consistency of the whole sample (*n* = 356) showed Cronbach's alpha values below those expected for the two domains.

Since the 1990s, the Center for Disease Control and Prevention (CDC) has developed reliability studies of the Youth Risk Behavior Survey (YRBS) questionnaire. More recent evidence from the original YRBSS framework further supports the reliability of this surveillance approach. A recent evaluation of the 2021 National Youth Risk Behavior Survey questionnaire conducted by the Centers for Disease Control and Prevention assessed test–retest reliability among 588 high school students. The authors reported that 96% of evaluated items demonstrated at least moderate reliability and 73% showed substantial or almost perfect reliability, with a mean Cohen's kappa coefficient of 0.68 (12). These findings are consistent with the excellent temporal stability observed in the present study and reinforce the robustness of standardized self-reported adolescent health-risk behavior measures.

A study carried out in Brazil to evaluate the psychometric properties of the items of the Youth Risk Behavior Survey (YRBS) in a sample of 902 university students demonstrated adequate internal consistency, with Cronbach's alpha = 0.770 for the global scale; these results were slightly greater than those found in our study. However, similarities with our study were found for an intraclass correlation coefficient (ICC) ≥ 0.75. The study concluded that the instrument seems to have satisfactory levels of criterion validity, internal consistency and temporal stability (11).

Overall, the reliability indicators observed in the present study were comparable to those reported in previous international adaptations of the YRBSS. Although internal consistency coefficients were slightly lower than those reported in Brazil and Iran, the intraclass correlation coefficients demonstrated excellent temporal stability, supporting the robustness of the Ecuadorian adaptation.

In a study conducted in Iran among 100 iranian adolescents aged 15-18 years, the reliability and validity of the psychometric properties of the self-administered Persian version of the 2009 Youth Risk Behavior Surveillance System (YRBSS) questionnaire and the Cronbach's *α* were 0.73 for intentional and unintentional injuries, 0.77 for tobacco use, 0.86 for alcohol and other drug use, and 0.79 for risky sexual behaviors; these results are slightly greater than those found in our study, but the ICCs that were 0.6-1 for moderate to excellent reliability were similar (13).

Regarding the internal consistency of the instrument, the results revealed a nuanced pattern across domains. The Total scale demonstrated an acceptable overall reliability (ω=0.780), suggesting that when aggregated, the items cohere adequately to measure the broader underlying construct. However, the domain-specific analyses showed lower internal consistency: **Social Aspects** yielded an omega of 0.680 (questionable/near acceptable), while **Health Aspects** recorded an omega of 0.661 (questionable) based on the thresholds established by Dunn et al. (8).

These findings warrant careful consideration. The lower omega values observed in the subdomains can be attributed to several methodological factors common in behavioral and public health research. First, the Health Aspects domain comprises a large and heterogeneous pool of items covering diverse health behaviors, conditions, and physical metrics. High item heterogeneity often deflates internal consistency indices because unidimensional models struggle to capture excessively broad domains uniformly. Second, certain social and health risk behaviors (such as specific substance use or rare adverse events) inherently exhibit low base rates or restricted variance in adolescent or general populations, which naturally depresses correlation matrices and reliability coefficients.

Consequently, while the global score supports the use of the instrument for broader evaluative purposes, researchers and practitioners should interpret the subscale scores particularly Health Aspects with caution, recommending future item refinement, cultural adaptation, or multidimensional structural modeling to improve psychometric robustness.

More recently, the reliability of the YRBSS has continued to be evaluated in different contexts and administration formats. A recent study conducted by the Centers for Disease Control and Prevention assessed the test–retest reliability of the 2021 National YRBS questionnaire among 588 high school students. The authors reported that 96% of the evaluated items demonstrated at least moderate reliability, and 73% showed substantial or almost perfect reliability, with a mean Cohen's kappa coefficient of 0.68 (12). These findings are consistent with the excellent temporal stability observed in the present Ecuadorian adaptation, supporting the robustness of self-reported adolescent health-risk behaviors when standardized procedures are applied

Recent evidence has confirmed the stability of YRBS measures in specific behavioral domains that are traditionally considered more susceptible to reporting bias. Among adolescents aged 16–18 years, test–retest analyses of substance use and sexual risk behavior items demonstrated moderate to substantial reliability across repeated administrations, suggesting that adolescents can provide relatively consistent reports of sensitive health behaviors when confidentiality is ensured (14).

Compared with these recent studies, the EYRBS demonstrated comparable or superior temporal reliability, with ICC values indicating excellent stability after a 15-day interval. However, differences in methodology should be considered, as previous YRBS reliability studies have mainly relied on Cohen's kappa for individual categorical items, whereas the present study additionally evaluated global temporal stability through intraclass correlation coefficients.

Recent studies have confirmed that sensitive behavioral domains included in the YRBSS can achieve acceptable temporal stability. Test–retest analyses of self-reported substance use and sexual risk behaviors among adolescents have demonstrated moderate to substantial reliability, suggesting that confidentiality procedures and appropriate administration strategies contribute to obtaining consistent responses for sensitive health behaviors (12). These findings are particularly relevant for the Ecuadorian adaptation because several included domains involve behaviors that may be affected by social desirability bias.

In another study conducted in Iran on a short version of the YRBS questionnaire among university students, the mean age was 23.54 ± 2.36 years. A Cronbach's alpha value greater than 0.7 was confirmed for all domains, which is slightly higher than the results of our study. The ICC was 0.735 (*P* = 0.007), similar to ours, which indicated the reliability of the questionnaire. The study concluded that the short-form questionnaire of the YRBS had the necessary psychometric properties to assess high-risk behaviors in young people (15).

### Limitations

Several limitations should be acknowledged. First, participants were recruited from educational institutions; therefore, adolescents who are not enrolled in formal education were not represented, which may limit generalizability. Second, although anonymity and confidentiality were ensured, self-reported measures may be influenced by recall bias and social desirability bias, particularly for sensitive behaviors such as substance use, violence, and sexual health. Third, structural validity was not assessed in the present study. In particular, the two-domain organization was conceptually defined rather than derived empirically through factor analysis. Future psychometric research should examine the structural validity of specific behavioral domains using analytical approaches appropriate to the multidimensional and conditional structure of the EYRBS. Fourth, some domains contained a limited number of items, which may have influenced internal consistency estimates. Finally, although culturally specific modifications improved the relevance of the questionnaire for Ecuadorian adolescents, additional studies using larger and more diverse samples are required to confirm the stability and validity of the adapted instrument. Although this study provides initial evidence of reliability, it did not evaluate structural validity through exploratory or confirmatory factor analysis, construct validity, McDonald's Omega, Composite Reliability, or other advanced psychometric properties. These analyses require larger samples and will be addressed in future studies. Finally, the virtual administration required adequate internet connectivity, which may have limited participation from adolescents living in more vulnerable settings.

Finally, while the global scale showed acceptable reliability, the domain-specific coefficients (Health Aspects and Social Aspects) fell into the questionable range (8), largely due to high item heterogeneity and restricted variance in sensitive behavioral indicators. Future research should consider item refinement and multidimensional modeling.

## Conclusions

The Ecuadorian adaptation of the Youth Risk Behavior Surveillance Survey demonstrated acceptable internal consistency and excellent test-retest reliability among Ecuadorian adolescents. This study provides initial evidence supporting the reliability of the Ecuadorian Youth Risk Behavior Survey following its cross-cultural adaptation. Although further psychometric evaluation is required to establish structural validity and other measurement properties, the EYRBS appears to be a promising instrument for monitoring health-risk behaviors among Ecuadorian adolescents. The availability of a reliable surveillance tool may facilitate the development of evidence-based interventions and public health strategies aimed at improving adolescent well-being in Ecuador.

## Data Availability

The raw data supporting the conclusions of this article will be made available by the authors, without undue reservation.
